# The Crystal Structure and RNA-Binding of an Orthomyxovirus Nucleoprotein

**DOI:** 10.1371/journal.ppat.1003624

**Published:** 2013-09-12

**Authors:** Wenjie Zheng, John Olson, Vikram Vakharia, Yizhi Jane Tao

**Affiliations:** 1 Department of Biochemistry and Cell Biology, Rice University, Houston, Texas, United States of America; 2 Department of Marine Biotechnology, University of Maryland Baltimore County, Institute of Marine and Environmental Technology, Baltimore, Maryland, United States of America; Institut Pasteur, France

## Abstract

Genome packaging for viruses with segmented genomes is often a complex problem. This is particularly true for influenza viruses and other orthomyxoviruses, whose genome consists of multiple negative-sense RNAs encapsidated as ribonucleoprotein (RNP) complexes. To better understand the structural features of orthomyxovirus RNPs that allow them to be packaged, we determined the crystal structure of the nucleoprotein (NP) of a fish orthomyxovirus, the infectious salmon anemia virus (ISAV) (genus *Isavirus*). As the major protein component of the RNPs, ISAV-NP possesses a bi-lobular structure similar to the influenza virus NP. Because both RNA-free and RNA-bound ISAV NP forms stable dimers in solution, we were able to measure the NP RNA binding affinity as well as the stoichiometry using recombinant proteins and synthetic oligos. Our RNA binding analysis revealed that each ISAV-NP binds ∼12 nts of RNA, shorter than the 24–28 nts originally estimated for the influenza A virus NP based on population average. The 12-nt stoichiometry was further confirmed by results from electron microscopy and dynamic light scattering. Considering that RNPs of ISAV and the influenza viruses have similar morphologies and dimensions, our findings suggest that NP-free RNA may exist on orthomyxovirus RNPs, and selective RNP packaging may be accomplished through direct RNA-RNA interactions.

## Author Summary

Orthomyxoviruses are a family of RNA viruses that include the various types of influenza viruses. The genome of orthomyxoviruses consists of multiple segments of negative-sense, single-stranded RNA molecules, each packaged in the form of rod-shaped, double-helical ribonucleoprotein (RNP) complexes. How different RNPs interact with each other to ensure specific genome packaging is a long-standing question and crucial to our understanding of orthomyxovirus replication and influenza virus gene reassortment. Our study of a fish orthomyxovirus, the infectious salmon anemia virus (ISAV), shows that its nucleoprotein (NP), which forms the protein scaffold backbone of the viral RNP, has a bi-lobular structure like the influenza virus NP. Because ISAV-NP forms stable dimers in solution, we were able to determine ISAV-NP RNA binding stoichiometry by biochemical assays, electron microscopy and dynamic light scattering. Our results indicate that each ISAV-NP binds ∼12-nt RNA, shorter than the 24–28 nts originally estimated for the influenza A virus based on population average. We propose that NP-free RNA exists on orthomyxovirus RNPs, and such RNA regions likely mediate specific RNP-RNP interactions during genome packaging. Further elucidation of the RNA-mediated RNP-RNP interactions will help us determine the molecular basis of gene reassortment by orthomyxoviruses including the influenza viruses.

## Introduction

Influenza viruses represent a serious public health concern due to their potential in causing widespread epidemics and pandemics. The genomes of the influenza A and B viruses are both comprised of eight segments of negative-sense RNA [1]. These eight gene segments are encapsidated in the form of rod-shaped, double-helical ribonucleoprotein (RNP) complexes in virions as well as in infected cells [2]. Each RNP contains a viral RNA, a heterotrimeric viral polymerase (consisting of PA, PB1, and PB2) and multiple copies of the viral-encoded nucleoprotein (NP) that bind viral RNA in a stoichiometric manner.

The segmented genome of influenza viruses offers a number of replication advantages such as gene recombination and transcriptional regulation, but at the same time it creates challenges for genome packaging. Once controversial, it is now widely accepted that the vRNPs of the influenza A viruses are specifically packaged into the virion, so that each particle contains eight unique segments of viral RNA (vRNA). Early evidence from defective-interfering RNAs (DI RNAs) and reverse genetics [3], [4] indicated that each gene segment possesses distinct packaging signals, and that unique sequences from both non-coding and coding regions at the 5′ and 3′ ends of all eight vRNA segments are essential for vRNA packaging [5]–[9]. Electron microscopy and tomography images showed that vRNPs in budding virions were organized in a distinct “7+1” pattern, with a long segment in the center surrounded by seven segments of various lengths at the periphery [10]–[13]. During virus assembly, vRNPs are aligned at the budding tip and interconnect with each other to form a supra-molecular assembly. Fluorescence *in situ* hybridization (FISH) analysis of single virus particles further confirmed that the eight unique RNPs are selectively incorporated into progeny virions and most virus particles contain only one copy of each of the eight RNP complexes [14].

Early electron microscopy studies of isolated influenza A virus RNPs revealed rod-shaped structures with a double-helical arrangement [15], [16]. Recently, cryo-EM reconstructions of the influenza A virus RNP have been calculated to ∼20 Å resolution [17], [18]. The double-helical stem region of the RNP shows a rise between two neighboring NP of ∼30 Å with five to six NP molecules per turn. Interaction between the two opposing arms of an RNP hairpin is solely mediated by the NP. Adjacent NP molecules on the same RNA strand interact through the extended NP tail loop. The putative RNA binding groove of the NP scaffold is exposed on the outer surface of the RNP.

Previous studies based on genome-wide stoichiometry calculations showed that there are ∼24–28 nts of RNA for each NP molecule [15], [19], [20]. In the absence of NP, vRNAs are able to mediate a comprehensive interaction network, with each vRNA segment interacting with at least one other vRNA partner, suggesting that RNPs are likely held together by direct base-pairings between packaging signals [12]. This model raises an intriguing question as to how the packaging signals are presented on the surface of the RNPs. Is vRNA maintained in an extended configuration devoid of any secondary structure by tight wrapping around the NP, or perhaps some NP-free RNAs exist in the RNP to allow vRNA-vRNA interaction?

To better understand the mechanism of influenza virus RNP packaging, we performed a series of structural and functional studies of the NP from the infectious salmon anemia virus (ISAV). ISAV is the first orthomyxovirus isolated from fish, and it (genus: *Isavirus*) is capable of causing wide-spread, severe anemia in Atlantic salmon and other salmonid species [21], [22]. Although their gene sequences are highly divergent, ISAV and influenza viruses share many similarities in their genomic, structural, morphological, and physiochemical properties. Here we determined the first crystal structure of an ISAV NP to 2.7 Å resolution. The crystal structure of ISAV-NP shows a head and a body domain that resemble those of the influenza A virus NP, despite the lack of sequence similarity. Using fluorescence anisotropy assays with synthetic RNA oligos, we showed that each ISAV-NP molecule binds to 12 nts of RNA, much shorter than the 24 nts originally estimated by genome-wide calculation for the related influenza A virus [15], [19], [20]. The 12-nt RNA binding stoichiometry is further supported by results obtained by electron microscopy and dynamic light scattering, as the addition of a 48-nt RNA to a solution of ISAV-NP led to the formation of NP tetramers or the dimerization of NP dimers. Our results thus suggest that a considerable amount of genomic RNA may exist in a NP-free state within the orthomyxovirus RNPs, and this free RNA may mediate the formation of high-order structures necessary for the specific recognition of the eight individual RNPs during virus assembly.

## Results and Discussion

### ISAV-NP forms stable dimers in solution

**Figure 1:**
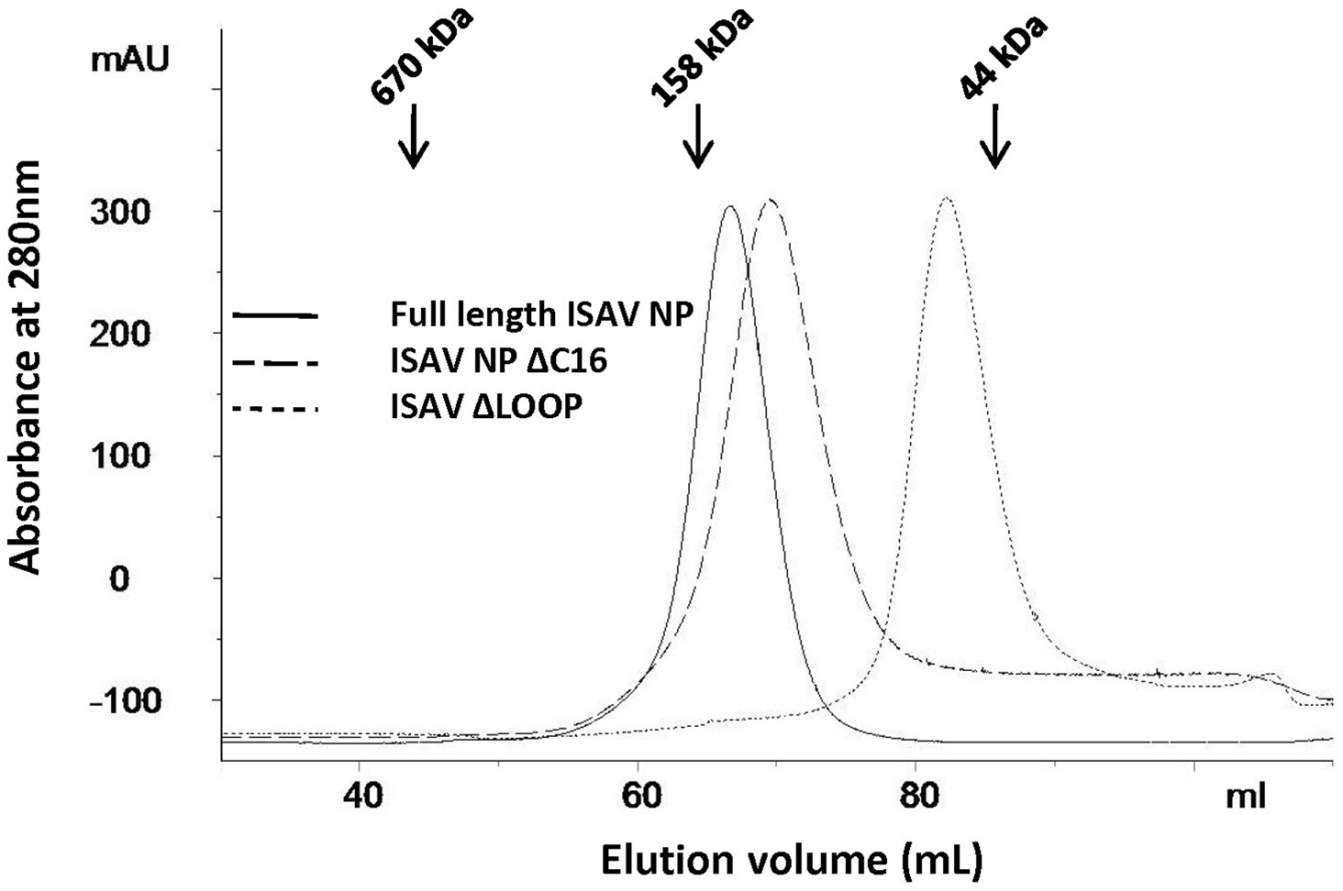
Gel filtration chromatograms for ISAV-NP proteins. The eluted positions of three protein standards are marked by arrows.

ISAV-NP was overexpressed in *E. coli* and purified as dimers with an approximate molecular weight of 140 kD, as estimated from the gel filtration chromatogram (Fig. 1). The ISAV-NP dimer was stable and did not dissociate under a wide range of conditions when tested *in vitro* (*e.g.* 0.01 to 10 mg/mL protein, NaCl ranging from 0.05 to 1 M, at 4 or 25°C for up to one month, etc). These unusual features of ISAV-NP make it an excellent system for studying NP RNA binding properties for the orthomyxoviruses, as the recombinant NPs of both influenza A and B viruses are known to form multiple oligomeric species [23]–[25]. Furthermore, influenza A virus NP oligomers and monomers undergo dynamic equilibrium in solution [23], and the addition of short RNA oligos led to the formation of larger oligomers possibly due to quaternary structure re-arrangement [24], [26]. RNPs from ISAV and influenza viruses exhibit similar double-helical morphologies in EM [15], [17], [18], [27].

### Crystal structure of ISAV-NP

Crystals of the full-length ISAV-NP were obtained, which only diffracted to ∼10 Å resolution. A number of N-terminal truncation mutants, including ΔN39, ΔN50, ΔN64, and ΔN84, and ΔN111, were subsequently cloned and expressed. Although ΔN50, ΔN64, and ΔN84 produced crystals, their diffraction was also poor. Visual inspection of the ISAV-NP sequence revealed a highly acidic C-terminal tail (*i.e.* ^600^EIEFDEDDEEEEDIDI^616^, with 12 out of the last 16 residues being either Glu or Asp); a feature that has been previously noted in many orthomyxovirus NPs. Without the last 16 residues, the ISAV-NP ΔC16 mutant also formed dimers, similar to the full-length protein (Fig. 1), and produced crystals that diffracted far better up to 2.7 Å resolution. The slight increase in the elution volume of the ISAV-NP ΔC16 mutant in gel filtration chromatography is likely due to changes in NP's radius of gyration or differences in protein-resin interaction.

**Figure 2:**
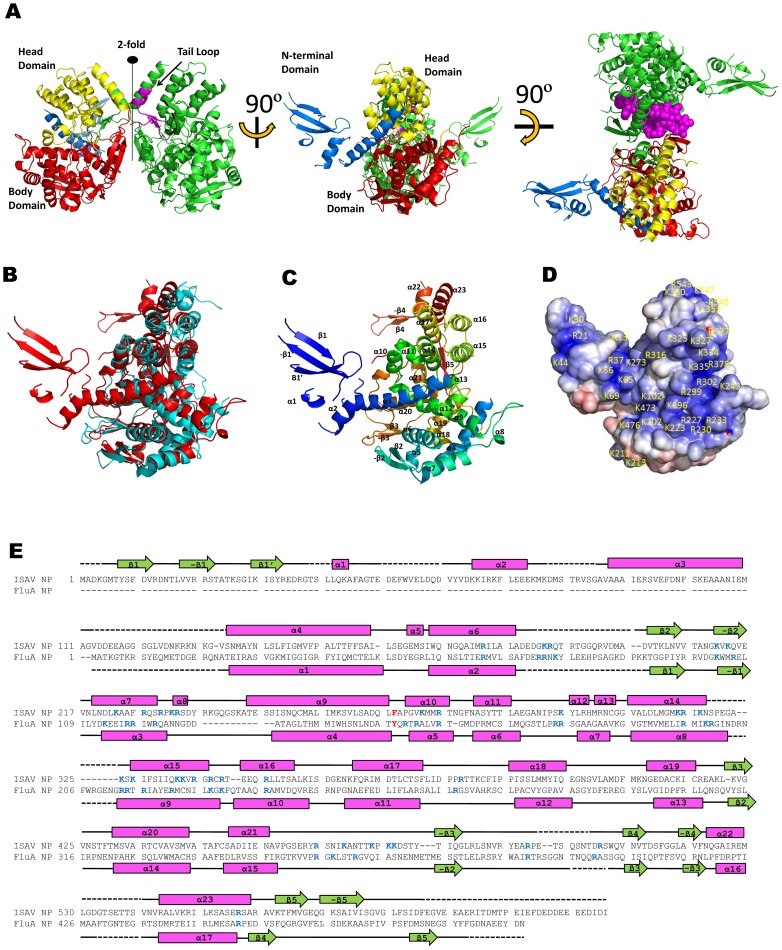
ISAV-NP crystal structure. (A) Three orthogonal views of an NP dimer. One subunit is colored in green, whereas the other subunit is colored by domains with blue for the N-terminal domain, yellow for the head, red for the body, and magenta for the tail loop. In the last panel, the magenta tail loop is shown by space-filling model to highlight the extensive interaction mediated by the tail loop. (B) ISAV-NP (red) superimposed onto the influenza A virus NP (cyan, PDB ID: 2IQH) [23]. (C) ISAV-NP monomer. The molecule is colored continuously from blue to red for the N-terminus to C-terminus, respectively. Secondary structural elements are numbered. (D) Calculated electronic potential for an NP monomer. Positively charged residues are highlighted. (B–D) are shown in the same orientation by viewing into the putative RNA binding groove, same as the middle panel in (A). (E) ISAV-NP secondary structure assignment. α-helices are shown by cylinders and the β-strands are represented by arrows. Conserved charged residues from the RNA binding groove are highlighted in blue. The conserved aromatic residue F274 in the groove is shown in red. The NP sequences from ISAV and the influenza A virus were manually aligned, based on tertiary structures.

The crystal structure of the ISAV-NP ΔC16 was determined by single-wavelength anomalous dispersion (SAD). The final model contains 495 amino acid residues in total, including residues 5–25, 27–38, 41–45, 61–75, 86–112, 133–182, 201–320, 325–497, 504–530, and 542–586. Each NP molecule can be divided into three domains: an N-terminal domain, a head domain, and a body domain (Fig. 2A). While the N-terminal domain is made up of a single polypeptide stretch containing the first 111 residues, the head and body domains are not colinear, with the head domain made from two excursions of the polypeptide chain (residues 272–380 and 494–558) out of the body domain. Structural comparison shows that the head and body domains of ISAV-NP closely resemble those of the influenza A virus NP, despite the lack of significant sequence similarity (Figure 2B, E).

Although there is only one NP in each crystallographic asymmetric unit, dimers are found around the crystallographic two-fold symmetry axes (Fig. 2A), which are presumably the same as those found in the solution (Fig. 1). The tail loop structure, which plays a critical role in influenza A virus NP oligomerization, is also found in ISAV-NP, although these two NP proteins form oligomers of different sizes (dimer vs. trimer). The tail loop of ISAV-NP (residues 504–530) folds into an extended β-hairpin that protrudes into a deep pocket in a neighboring subunit to mediate dimer formation. The interaction made by the tail loop is primarily hydrophobic in nature. A single charged residue Asp513 is found near the tip of the tail loop. The side chain of Asp513 hydrogen bonds to four main-chain nitrogens in the opposing peptide strand from the same tail loop, thus stabilizing the formation of the β-hairpin within the tail loop. The ISAV-NP dimer interface is ∼3520 Å^2^ in size, of which a 1518 Å^2^ area is mediated by the tail loop alone. Deletion of the tail loop (ΔLOOP or Δ504–530) resulted in the formation of NP monomers only (Fig. 1), which suggests an important oligomerization function for the tail loop structure in orthomyxoviruses in general.

### The ISAV-NP RNA binding groove

Analogous to the influenza A virus NP, there is a potential RNA-binding groove between the head and body domains of the ISAV-NP. The groove is positively charged according to its calculated electrostatic potentials, and is covered with a large number of basic amino acid residues, which presumably interact with the negatively charged phosphodiester backbone of bound RNA (Fig. 2D, Fig. S1 – Supporting Information). All of these basic residues, except those from the N-terminal domain, are also found in the influenza A virus NP, although their positions may slightly shift on the primary sequence (Fig. 2E). The aromatic residue F274, analogous to Y148 from the influenza A virus NP [23], is found at one end of the RNA-binding groove, and may mediate nucleotide base stacking.

To test whether ISAV-NP binds RNA in the same manner as the influenza A virus NP, we made two double mutants: K185A/R186A and K296A/R299A. These two pairs of basic residues are from two flexible loops in the potential RNA binding groove that are partially disordered in the ISAV-NP structure. At equivalent positions in the influenza A virus NP, R74/R75 and R174/R175 were found to play critical roles in RNA binding [24]. As expected, mutations of these residues resulted in a dramatic decrease in the RNA binding affinity, as the *K_d_* changed from 24.5 nM for the wild-type *(wt)* protein to 355 nM and 472 nM for the K185A/R186A and K296A/R299A mutants, respectively (Supporting Information, Fig. S2). This result indicates that NPs of both ISAV and the influenza A virus likely bind RNA with their positively charged groove.

### ISAV-NP N-terminal domain and C-terminal tail

**Figure 3:**
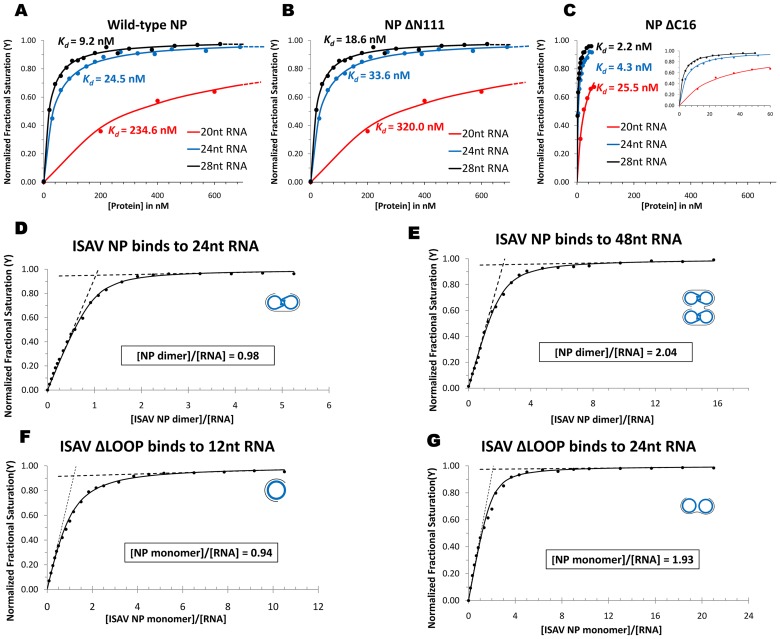
ISAV-NP RNA binding. (A–C) RNA binding affinity measurements for the *wt* NP (A), ΔN111 (B) and ΔC16 (C). FA was performed using three RNA oligos each containing 20, 24, and 28 nts. The binding curves are plotted using the same protein concentration range. For the ΔC16 mutant, an inset is added in (C) to provide a more spread-out view at lower protein concentrations. Fully saturated curves for the 20-nt RNA are shown in Fig. S3 in Supporting Information. (D–G) RNA binding stoichiometry measurements for the *wt* NP and the ΔLOOP monomeric mutant. NP:RNA complexes assembled in each experiments are schematically shown with blue handcuffs for NP dimers and grey curves for RNA molecules.

The N-terminal domain of ISAV-NP is a unique structural feature that was not observed in the crystals structures of either the influenza A or influenza B virus NP. Consisting of three anti-parallel β-strands and two α-helices, the ISAV N-terminal domain adopts an extended shape and interacts with the rest of the structure mainly through the long α-helix made of residues 86–112. This large α-helix partially covers the potential RNA binding groove in between the head and body domain. Deleting the entire N-terminal domain had little effect on the equilibrium-state RNA binding, as *wt* and ΔN111 NP exhibited very similar RNA binding affinities (*K_d_* = 24.5 nM for *wt* vs. 33.6 nM for ΔN111, using a 24-nt RNA) (Fig. 3, see the section below for more details). Nevertheless, it is plausible that the N-terminal domain may regulate the dynamics of the NP RNA binding. For instance, it may help to attract RNA substrates and/or serve as a cap to prevent the dissociation of RNA prior to being locked in a bound position. We speculate that the long α-helix from the N-terminal domain may need to be displaced from its current location upon RNA binding. The inherent structural flexibility of the N-terminal domain is evident from its high temperature factors, averaging 85.0 Å^2^ for the N-terminal domain, as compared to 54.8 Å^2^ for the rest of the structure.

A multi-sequence alignment based on secondary structure shows that a N-terminal domain is consistently found in every orthomyxovirus NP, but it is highly variable in both size and sequence (*e.g.* 21aa for influenza A virus, 72aa for influenza B virus, and 111aa for ISAV). Furthermore, the N-terminal domain is structurally disordered in both the influenza A and influenza B virus NPs [23]–[25]. In influenza A virus, the first 20 amino acid region contains a phosphorylation site and a nuclear localization signal (NLS) that are important for the intracellular trafficking of the NP [28], [29]. Such functions have not been attributed to the ISAV N-terminal domain. Although two monopartite nuclear localization signals ^230^RPKR^233^ and ^473^KPKK^476^ have been identified in ISAV-NP [30], both were mapped to the surface of the body domain. The location of the N-terminal domain suggests that it would be situated on the exterior of assembled RNPs, and therefore may help to mediate interactions with the viral matrix protein and/or NS2 to facilitate the intracellular trafficking of viral RNPs.

At the C-terminal end of the ISAV-NP polypeptide is a highly acidic C-terminal tail that was excluded from our NP construct used for crystal structure determination (see discussions above). The last ordered residue in the crystal structure of ISAV-NP ΔC16 (consisting of residues 1 to 600) is residue 586, suggesting that the entire polypeptide region after residue 586 is likely to be disordered. We have shown that removing the last 16 residues of the ISAV-NP resulted in an enhanced RNA binding affinity (i.e. ∼6-fold increase for a 24-nt RNA) (Fig. 3A, C, see the section below for more details). The exact functional role of the NP C-terminal tail during virus replication remains unclear, and it is possible that the C-terminal tail may assume a different conformation at other functional states. For the influenza A virus NP, the last eight residues are also negatively charged. These last residues of the influenza A virus NP have been found to assume three different structural conformations: (1) structurally disordered [23]; (2) structurally ordered and bound to the putative RNA binding groove [26], [31]; and (3) structurally ordered but pointing away from the RNA binding groove [24]. It was proposed that the acidic C-terminal tail of the influenza A virus NP may function to negatively regulate NP RNA binding activity [26], [31]. Here, our finding that the ISAV-NPΔC16 mutant has a stronger RNA binding affinity appears to support this hypothesis.

### ISAV-NP RNA binding

Using synthetic RNA oligos and fluorescence polarization assays, we first measured the RNA-binding affinity of three NP variants, *wt* NP, NP ΔN111, and NP ΔC16. ΔN111 lacks the N-terminal domain, so its structure is comparable to that of the influenza A virus NP. Because genome-wide stoichiometry estimation revealed that each influenza A virus NP binds to 24 nts of RNA [19], the oligonucleotide (AC)_12_ (consisting of 12 AC repeats) was chosen for our initial assays. Interestingly, *wt* NP and ΔN111 exhibited similar RNA-binding affinities (Fig. 3A and B), suggesting that the N-terminal domain is not actively involved in equilibrium-state RNA binding. On the other hand, NP ΔC16 exhibited a much stronger RNA binding affinity than *wt* NP, indicating the acidic C-terminal tail negatively regulates NP-RNA binding. When the RNA oligo was shortened from 24 nts to 20 nts, the binding affinity of NP dropped sharply with a ∼10-fold increase in *K_d_* (Fig. 3A). On the other hand, when the size of the RNA oligo was increased from 24 nt to 28 nt; there was little change in NP-RNA binding affinity (Fig. 3A). Similar to the full-length NP, RNA size has little effect on the *K_d_*'s for binding to the ΔN111 and ΔC16 mutants, even though the latter shows a ∼10-fold higher intrinsic affinity (Fig. 3B and C). Therefore, the 24 nt-long RNA has saturated the NP-RNA binding site, and the small increase in binding affinity from 24-nt to 28-nt RNA was probably due to non-specific binding. The same trend was also observed when DNA oligos ranging from 15 to 30 nts long were used for binding (Supporting Information, Fig. S4).

To determine whether each 24-nt RNA binds to a single NP subunit or to both subunits in a NP dimer, we used the same fluorescence polarization assay to quantify binding stoichiometry. To ensure stoichiometric binding, we adjusted the experimental conditions such that the total concentration of RNA was significantly above the binding dissociation constant *K_d_* (see Online Methods). When the *wt* NP was titrated in the solution, the fraction of bound RNA, as noted from millipolarization (mP) signal, first increased linearly and then gradually approached saturation (Fig. 3D). Using either the inflection point extrapolation or direct fitting of the general titration equation, we arrived at the same conclusion that each 24-nt RNA binds to an NP dimer. Subsequently, a similar experiment using a 48-nt RNA gave a stoichiometry of two NP dimers per 48-nt long RNA (Fig. 3E), consistent with our earlier result of each NP monomer binding to ∼12 nts of RNA.

To rule out the possibility that one NP subunit binds 24 nts of RNA and that RNA binding by one NP subunit allosterically inhibits RNA binding by the other subunit in the NP dimer, a monomeric ΔLOOP mutant (Fig. 1) was used for RNA binding assays as described above. Circular dichroism spectra of the ΔLOOP mutant were very similar to that of the *wt* NP trimer, indicating similar overall secondary structure contents (Supporting Information, Fig. S5). For the influenza A virus, the NP ΔLOOP mutant has a tertiary structure that is nearly identical to the *wt* NP except for the lacking of the tail loop [31]. A monomeric mutant with a single amino acid substitution (*i.e.* R416A) in the tail loop of the influenza A virus NP was found to undergo some structural rearrangements in the C-terminal part of the polypeptide, but the potential RNA binding groove remains largely intact [26].

As expected, the RNA binding affinity of the ISAV-NP ΔLOOP monomer was very similar to that of the *wt* NP for a 12-nt long RNA, which supposedly contains only one NP binding motif (*K_d_* = 488.4 nM for *wt* vs. 1076.1 nM for ΔLOOP NP). However, the protein binding stoichiometries of the 12-nt and 24-nt RNAs were unexpectedly different. While each 12-nt RNA bound to one ΔLOOP NP monomer, each 24-nt RNA recruited two NP ΔLOOP monomers when fully saturated (Fig. 3F, G). Therefore, results obtained from the ΔLOOP mutant are consistent with our earlier conclusion based on the *wt* NP dimer.

**Figure 4:**
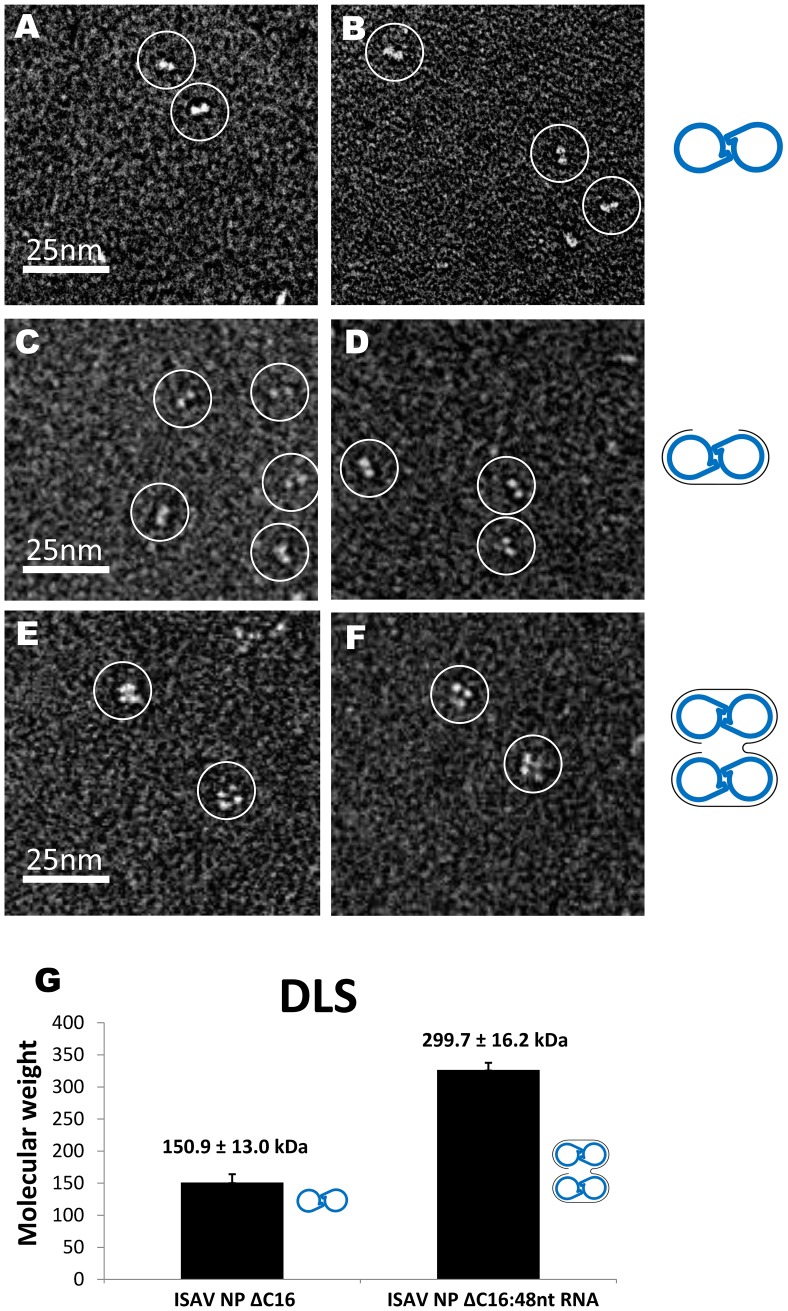
ISAV-NP:RNA complexes. (A–F) EM images of the free NP (A, B), NP bound to a 24-nt RNA (C, D), and NP bound to a 48-nt RNA (E, F). (G) Molecular weights of NP and NP:RNA complexes as determined by DLS. Molecular interpretations were made for each figure with blue handcuffs for the NP dimers and grey curves for RNA molecules.

We then used negative-staining electron microscopy (EM) to confirm the RNA-binding stoichiometry by measuring the number of NP molecules associated with each RNA molecule. As shown in Fig. 4, ISAV-NP formed dimers in the absence of RNA, with each NP molecule assuming the shape of a 4×5 nm^2^ oval (Fig. 4A, B). When the 24-nt RNA was added to NP at the 1∶2 molar ratio (*i.e.* one RNA per NP dimer), the same dimeric assemblies were observed (Fig. 4C, D). However, when the 48 nt-RNA were added to ISAV-NP at the 1∶4 ratio (*i.e.* one RNA for four NP subunits), we observed mostly tetramers, which were presumably formed by simultaneous binding of two NP dimers to one 48-nt RNA (Fig. 4E, F).

Similar results were also obtained from size measurement using dynamic light scattering (DLS) (Fig. 4G). Assuming that ISAV-NP adopts a roughly globular shape, the molecular weight of the *wt* NP dimer was 150.9±13.0 kD by DLS measurement. The calculated molecular weight of a dimer is 136.5 kD, and the slight difference could be explained by the non-globular shape of the dimer. When the 48-nt RNA was added to the NP sample, the molecular weight of the complex was increased to 299.7±16.2 kD, which matches closely to the calculated molecular weight of two ISAV-NP dimers binding to a 48-nt poly(C) RNA (2×136.5+14.8 = 287.8 kD). Our results from EM and DLS once again confirm that one ISAV-NP molecule binds to a 12-nt RNA.

### Functional implication of the orthomyxovirus NP-RNA binding

EM images of ISAV RNPs released from virions showed double-helical structure with similar dimensions compared to those from the influenza A viruses. For instance, it was reported that the longest RNPs of ISAV were ∼120 nm long [27], very close to the maximum length of ∼110 nm measured for the influenza A virus RNPs [15]. ISAV contains eight gene segments, with the longest one, which encodes PB2, containing ∼2.4 kb of nucleotides. This is only slightly larger than the longest RNA segment of the influenza A virus, which is ∼2.3 kb in length. Therefore, considering that ISAV and the influenza A virus have similarly sized RNPs as well as RNAs, we expect that the NP RNA binding stoichiometry should be similar for these two viruses. Hence, each ISAV-NP is likely associated with ∼24–28 nts of RNA in the infectious virion, as previously determined for the influenza A viruses based on population average.

Since only the head and body domain of ISAV-NP affect equilibrium-state RNA binding, ISAV-NP should serve as a valid model for studying the NP-RNA binding for other orthomyxoviruses including the influenza viruses. We conclude that each ISAV-NP monomer, and possibly each influenza A virus NP, bind ∼12 nts of RNA. This stoichiometry of binding is also supported by the observation that the potential RNA-binding groove of the ISAV and the influenza A virus NP is ∼60 Å long. Assuming that bound nucleotides are ∼5 Å apart (*i.e.* based on single-stranded RNAs bounds to nucleocapsids from the tobacco mosaic virus and also rhabdoviruses), each NP molecule should bind no more than ∼12 nts of RNA, consistent with our stoichiometric measurements. It has also been noted by Jennings et al. that the length of 22–26 nts exceeds the dimension of NP, so RNA coiling or looping may be necessary for RNP assembly [32]. Indeed, RNase digestion of an influenza A virus RNPs and mini-RNPs produced RNA fragments of ∼15–18-nt in size [33], [34], indicating that the size of RNA bound by each NP should be shorter than 15–18 nts long.

The cryo-EM reconstruction of influenza A virus RNPs shows that NP molecules follow a regular double-helical arrangement, with the potential RNA binding groove facing outward [17], [18]. At the current resolution, RNA densities cannot yet be identified in the EM maps. Compared to NP dimer or longer unconstrained NP polymers, the double-helical stem region of the RNP [17], [18] shows a wider separation between adjacent NP molecules along the same NP strand and thus may require short RNA linkers made of up to four extra bases. However, even with such linkers considered, RNA would still follow a highly convoluted path if 24 nts of RNA are modeled for each NP molecule in a RNP [17]. Moreover, if we assume that the double-helical RNP is converted from a linear NP-RNA polymer precursor during assembly, then double-helical RNPs and linear NP-RNA polymers should have similar NP RNA binding stoichiometry. Therefore, we believe that the geometric difference between the helical RNP and the unconstrained NP-RNA polymer cannot completely compensate for the substantial difference in NP RNA stoichiometry (*i.e.* ∼24 nts vs. ∼12 nts for each NP).

Based on our stoichiometry measurements and the rationale as stated above, we speculate that a fraction of the orthomyxovirus (*e.g.* ISAV, influenza A viruses, etc) genomic RNA likely exists in a NP-free state in the context of RNP. Such NP-free RNA regions are probably rich in secondary or tertiary structures, as it has been shown that double-stranded RNAs bind less favorably to NP compared to single-stranded RNAs. We envision that there is an uneven distribution of NP along the viral RNA that varies depending on the secondary/tertiary structure of the RNA. Because nascent v/cRNA molecules are encapsidated by NP co-transcriptionally, we assume that only local RNA structures occur in RNPs. Although little is known about the details of such RNA structures, presumably they need to be stable enough to resist denaturation by NP binding, as it has been reported that the influenza A virus NP was able to melt the viral RNA panhandle structure [35]. These RNA structures likely play a critical role in the packaging of viral genome during the assembly process, possibly by forming specific higher-order structures. These structures would allow the eight different RNPs to recognize each other and form the “7+1” array previously seen in EM images of budding influenza A virus particles [10]–[13], [36]. Thus, the 2-nm thick filamentous masses connecting neighboring RNPs in budding influenza A viruses are likely RNA structures formed by packaging signals [13]. Because the RNA packaging signals are usually found near both ends of the vRNA molecules [37], each RNP may interact with at least two neighboring RNPs to build up a network of interactions that ensure specific genome packaging.

Crystal structures of NP proteins or NP-RNA complexes have been reported for a number of other negative-sense, single-stranded RNA viruses including members from the rhabodovirus, paramyxovirus, bornavirus, arenavirus and bunyavirus families [38]–[53]. Among negative strand RNA viruses, rhabodovirus, paramyxovirus, filoviru and bornavirus have a non-segmented genome, whereas arenavirus, bunyavirus and orthomyxovirus have a genome comprised of multiple RNA segments. For non-segmented negative strand RNA viruses, it has been shown by crystal structure and/or genetic evidence that their RNPs are strictly stoichiometric complexes, with NP proteins distributed evenly along the NP-RNA complexes; whereby vRNA is completely unwound and protected by the NP [38]–[41], [54], [55]. For rhabodovirus, paramyxovirus, and filovirus, each of their NP proteins binds 9, 6/7, and 6 nucleotides of RNA, respectively [39]–[41], [54], [55]. Such properties are consistent with the function of these RNPs, as the genomic sequences of non-segmented negative-sense RNA viruses are not expected to mediate RNP packaging into viral particles. Compared to NPs from non-segmented, negative-sense RNA viruses, orthomyxovirus NPs have a different topology and are relatively large in size. With NP-free RNA exposed on the outer surface, the structural organization of the orthomyxovirus RNP is optimally suited for multi-segment RNA packaging. Future efforts in identifying RNA secondary structure will likely reveal molecular determinants for RNP recognition and genomic RNA reassortment for the influenza viruses and other orthomyxoviruses. It will also be of great interest to identify molecular determinants used by arenaviruses and bunyaviruses to facilitate specific RNP packaging, should such a specific genome package mechanism exist in these segmented RNA viruses as well.

## Materials and Methods

### 1. Cloning

The full-length ISAV-NP (aa 1–616), the ΔC16 NP (aa 1–600), and the ΔN111 NP (aa 112–616) were cloned with an N-terminal 6×His tag. The polymerase chain reactions were performed using the *PfuUltra™* high-fidelity DNA polymerase (Stratagene). PCR products were digested with NcoI and XhoI (New England Biolabs) for 2 hours, and ligated into the pET28b+ vector for expression in Rosetta 2 *E. coli* cells (DE3) (Novagen). For the ISAV ΔLOOP mutant, a short linker sequence “Gly-Gly-Gly-Gly-Ser” was used to replace the “tail-loop” (aa 504–530).

### 2. Expression, purification, and crystallization

The expression of NP proteins was induced with 0.1 mM IPTG at 15°C for 24 hours after the cell density had reached an OD_600 nm_ of 0.8. SeMet-substituted NP proteins were expressed in M9 minimal medium supplemented with seleno-methionine (ACROS organics). Cells were collected and sonicated in lysis buffer containing 300 mM NaCl, 5 mM Imidazole, 10% glycerol (v/v), and 50 mM Tris·HCl, pH 7.5. The lysate was centrifuged at 40,000× *g* for 30 min and the supernatant was used for further purification. Recombinant NP proteins were purified using a HisTrap™ HP Column, a HiTrap™ Heparin HP Column, and finally a Superdex™ 200 Column (GE Healthcare). Purified proteins were at least 95% pure according to Coomassie-stained SDS–polyacrylamide gels. All proteins were concentrated to 10 mg/ml and stored at 4°C.

Crystals of ISAV-NP ΔC16 were obtained from a protein-ssDNA complex containing the oligo dC_18_. Although the DNA oligo could not be seen in the electron density, its presence was required for crystal formation. The best ΔC16 crystals were grown at 10°C for two weeks using the sitting-drop vapor diffusion method with the drop made of equal volume of protein solution (10 mg/ml in 200 mM NaCl, 10% glycerol, and 50 mM Tris·HCl, pH 7.5) and well solution (containing 0.8 M lithium chloride, 0.1 M sodium citrate, pH 4.0, and 20% PEG 6000). To improve crystal diffraction, an additional dehydration step was performed by transferring crystals to a mother liquor containing 0.8 M lithium chloride, 0.1 M sodium citrate, pH 4.0, 9% PEG 6000 and 20% PEG 400. After 24 hours, the crystals were directly frozen in liquid nitrogen.

### 3. Data collection, phase determination, model building, and refinement

Diffraction data were collected at the Beamline 4.2.2 at the Advanced Light Source, Berkeley. All diffraction data were processed using the program HKL2000 [56]. The structure was determined by single-wavelength anomalous diffraction (SAD). The heavy atom sites and experimental phases were determined by the AutoSol Wizard in the PHENIX software suite [57]. The protein model was built with Autobuild (PHENIX) and COOT [58], and refined with phenix.refine. All the structure figures were prepared using the program PyMOL unless otherwise specified (The PyMOL Molecular Graphics System, Version 1.2r3pre, Schrödinger, LLC). The coordinates have been deposited at the Protein Data Bank (PDB ID 4EWC)

### 4. Fluorescence polarization assays

5′-fluorescein-labeled RNA oligos were purchased from Thermo Fisher Scientific and Sigma-Aldrich. The sequences of the RNA oligos used are simple “AC” repeats (*e.g.*, a 12mer RNA has the sequence of 5′–(AC)_6_–3′) to avoid potential secondary structures that may interfere with protein binding.

For the affinity measurements shown in Fig. 3A–C, our binding solutions contained 100 mM NaCl, 25 mM Tris, pH 7.5, 10% glycerol, and fluoresceinated RNA at 0.2 nM or higher (at least 10-fold lower than *K_d_*). NPs were titrated into the binding solution until the millipolarization (mP) stabilized. All of the experiments were carried out at 25°C. The data were plotted and analyzed by using the equation P = {(P_bound_−P_free_)[protein]/(*K_d_*+[protein])}+P_free_, where P is the polarization measured at a given total protein concentration, P_free_ is the initial polarization of fluorescein-labeled RNA without protein bound, P_bound_ is the maximum polarization of RNA when all are bound by protein, and [protein] is the protein concentration. The free and total protein concentrations were assumed to be equal since the *K_d_* was at least 10-fold higher than the concentration of fluorescein-labeled RNA. The hyperbolic curves were fitted by nonlinear least-squares regression analysis assuming a bimolecular model of pseudo first order such that the *K_d_* values represent the protein concentrations at which half of the ligands are in the bound-state.

For the FA experiments used to determine the binding stoichiometry, the assay conditions were identical to those used for the binding affinity determination, except that the RNA concentration was increased to 60 nM and the NaCl concentration was reduced to 50 mM. These adjustments were made to ensure that the RNA concentration was at least 20-fold higher than the *K_d_*. The titration curves showed a linear increase (first phase) in millipolarization until RNA-binding sites were saturated (second phase). Data points belonging to each phase were separately fitted by linear least-squares regression analysis to produce the inflection point, at which point the RNAs were stoichiometrically bound with protein.

### 5. Electron microscopy

The ISAV-NP ΔC16:48 nt-RNA sample was prepared by mixing the protein with the 48 nt-ssRNA at a 4∶1 molar ratio in 100 mM NaCl and 25 mM HEPES, pH 7.5. The ΔC16:24 nt-RNA complex was prepared by mixing protein with RNA at a molar ratio of 2∶1. Both samples were incubated at 25°C for 30 min and purified by gel filtration before use.

Carbon-coated copper grids (Electron Microscopy Sciences) were glow discharged at 5 mA for 1 min. 3 uL of protein samples at 0.02 mg/mL was added onto glow-discharged copper grids, washed twice with distilled water, and stained with freshly prepared 1.5% uranyl acetate solution for 1 min. Images were taken with a JEOL 2010 electron microscope (JEOL USA, Inc.) operated at 100 KeV using a magnification of 40,000×.

### 6. Dynamic light scattering

The ISAV-NP ΔC16:48 nt-RNA sample was prepared in the same manner as for EM. DLS experiments were performed using a DynaPro99 Dynamic Light Scattering system from Wyatt. All of the samples were centrifuged at 22,000×*g* for 30 min to remove large aggregates prior to the experiments. The DLS experiments were carried out at 25°C in 100 mM NaCl and 25 mM HEPES, pH 7.5. For each sample, 20 molecular weight readouts were calculated with each being the average of five individual measurements. The 20 readouts were further averaged to obtain the mean and standard deviation of the sample molecular weights.

## Supporting Information

Figure S1 Calculated electronic potential for an ISAV-NP monomer. Positively charged residues are highlighted. The diagram on the left is the same as the one shown Figure 2D.
(DOCX)

Figure S2 RNA binding by *wt* and mutant ISAV-NP. RNA binding affinity measurements for the *wt* NP and its two double mutants, K185A/R186A and K296A/R299A, were performed by FA using a 24-nt RNA.
(DOCX)

Figure S3 ISAV-NP RNA binding. RNA binding affinity measurements for the *wt* NP, ΔN111 and ΔC16 were performed by FA using a 20-nt RNA oligo.
(DOCX)

Figure S4 ISAV-NP DNA binding. DNA binding affinity measurements for the *wt* NP were performed by FA using four poly(C) oligos ranging from 15 to 30 nucleotides long. (A) and (B) are plotted against a narrower and broader X-axes, respectively.
(DOCX)

Figure S5 Far-UV CD Spectra of ISAV NP. All three NP proteins, including the *wt* NP (blue), ΔLOOP (red) and ΔC16 (green) were kept at 5 µM concentration. Protein buffer was 200 mM potassium phosphate pH 7.5. Measurements were done at room temperature.
(DOCX)

Table S1 Crystallographic data statistics.
(DOCX)
