# Supplementary material for: The Crystal Structure and RNA-Binding of an Orthomyxovirus Nucleoprotein
Source: PLoS Pathog. 2013 Sep 12;9(9):e1003624. doi: 10.1371/journal.ppat.1003624 (PMC3771910; doi:10.1371/journal.ppat.1003624)
Supplement: Figure S1 — Calculated electronic potential for an ISAV-NP monomer. Positively charged residues are highlighted. The diagram on the left is the same as the one shown Figure 2D. (DOCX) [file ppat.1003624.s001.docx]

**
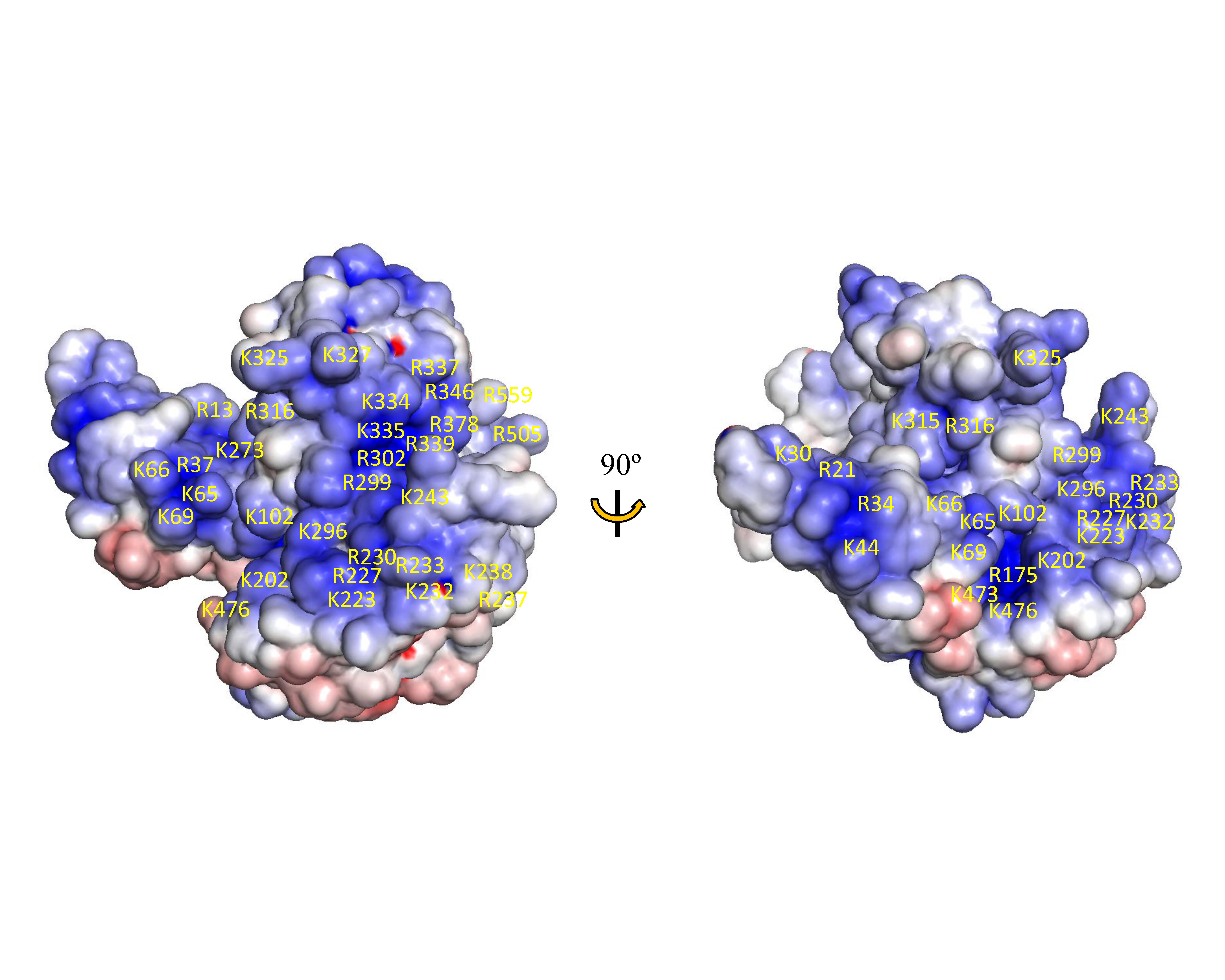
**

**Figure S1**. Calculated electronic potential for an ISAV-NP monomer. Positively charged residues are highlighted. The diagram on the left is the same as the one shown Figure 2D.
