# Supplementary material for: The Crystal Structure and RNA-Binding of an Orthomyxovirus Nucleoprotein
Source: PLoS Pathog. 2013 Sep 12;9(9):e1003624. doi: 10.1371/journal.ppat.1003624 (PMC3771910; doi:10.1371/journal.ppat.1003624)
Supplement: Figure S2 — RNA binding by wt and mutant ISAV-NP. RNA binding affinity measurements for the wt NP and its two double mutants, K185A/R186A and K296A/R299A, were performed by FA using a 24-nt RNA. (DOCX) [file ppat.1003624.s002.docx]

**Figure S2**. RNA binding by *wt* and mutant ISAV-NP. RNA binding affinity measurements for the *wt* NP and its two double mutants, K185A/R186A and K296A/R299A, were performed by FA using a 24-nt RNA.
