# Supplementary material for: The Crystal Structure and RNA-Binding of an Orthomyxovirus Nucleoprotein
Source: PLoS Pathog. 2013 Sep 12;9(9):e1003624. doi: 10.1371/journal.ppat.1003624 (PMC3771910; doi:10.1371/journal.ppat.1003624)
Supplement: Figure S3 — ISAV-NP RNA binding. RNA binding affinity measurements for the wt NP, ΔN111 and ΔC16 were performed by FA using a 20-nt RNA oligo. (DOCX) [file ppat.1003624.s003.docx]

**Figure S3**. ISAV-NP RNA binding. RNA binding affinity measurements for the *wt* NP, ΔN111 and ΔC16 were performed by FA using a 20-nt RNA oligo.
