# Supplementary material for: The Crystal Structure and RNA-Binding of an Orthomyxovirus Nucleoprotein
Source: PLoS Pathog. 2013 Sep 12;9(9):e1003624. doi: 10.1371/journal.ppat.1003624 (PMC3771910; doi:10.1371/journal.ppat.1003624)
Supplement: Figure S4 — ISAV-NP DNA binding. DNA binding affinity measurements for the wt NP were performed by FA using four poly(C) oligos ranging from 15 to 30 nucleotides long. (A) and (B) are plotted against a narrower and broader X-axes, respectively. (DOCX) [file ppat.1003624.s004.docx]

**
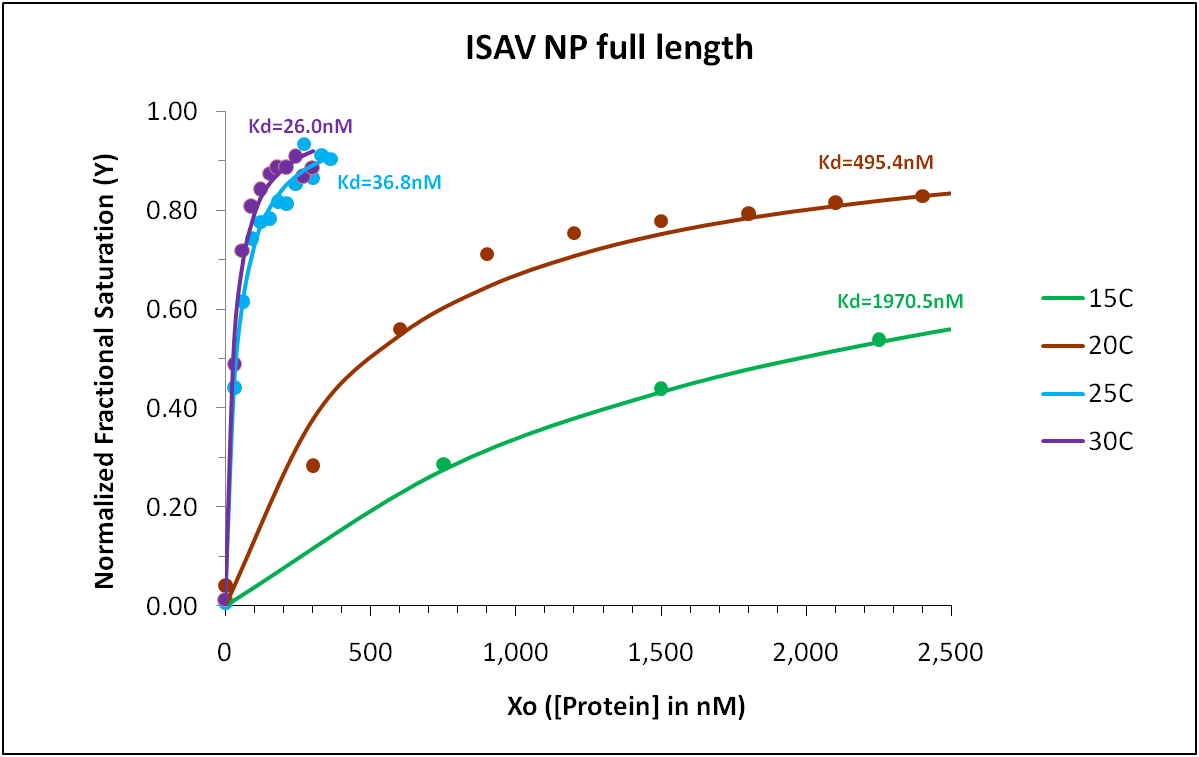

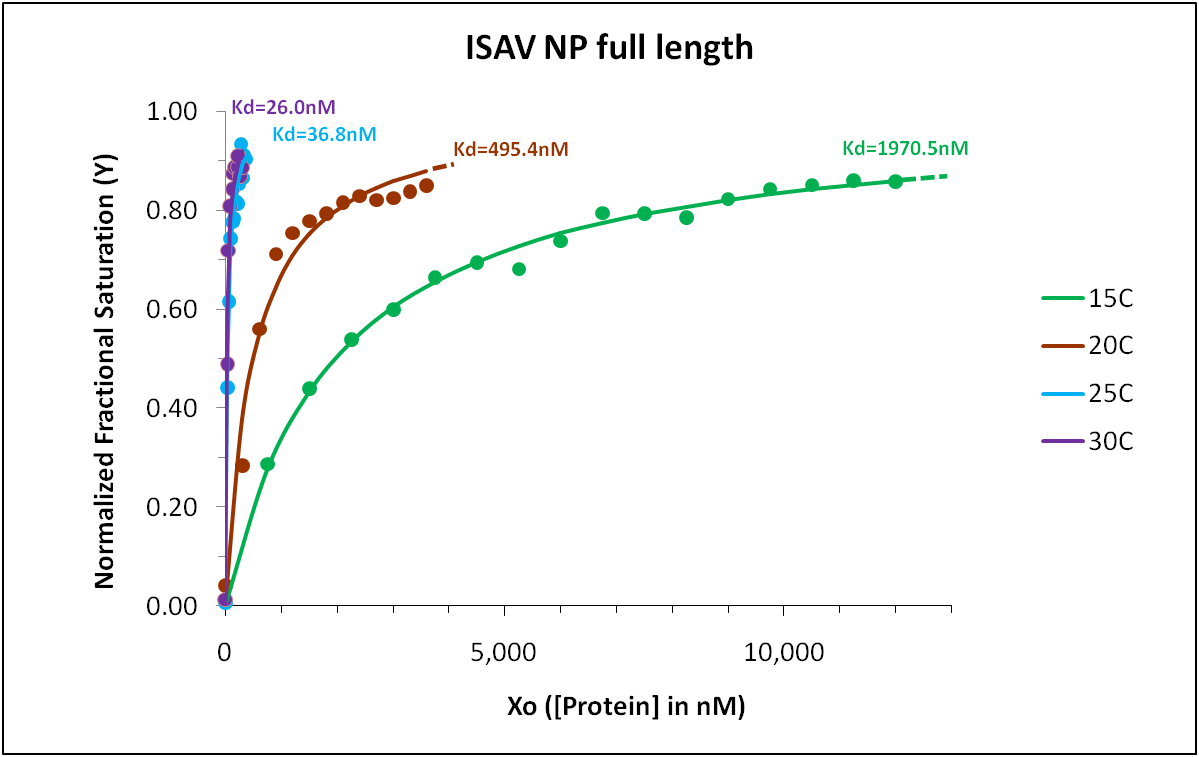
**

**B**

**A**

**Figure S4**. ISAV-NP DNA binding. DNA binding affinity measurements for the *wt* NP were performed by FA using four poly(C) oligos ranging from 15 to 30 nucleotides long. (A) and (B) are plotted against a narrower and broader X-axes, respectively.
