# Supplementary material for: The Crystal Structure and RNA-Binding of an Orthomyxovirus Nucleoprotein
Source: PLoS Pathog. 2013 Sep 12;9(9):e1003624. doi: 10.1371/journal.ppat.1003624 (PMC3771910; doi:10.1371/journal.ppat.1003624)
Supplement: Figure S5 — Far-UV CD Spectra of ISAV NP. All three NP proteins, including the wt NP (blue), ΔLOOP (red) and ΔC16 (green) were kept at 5 µM concentration. Protein buffer was 200 mM potassium phosphate pH 7.5. Measurements were done at room temperature. (DOCX) [file ppat.1003624.s005.docx]

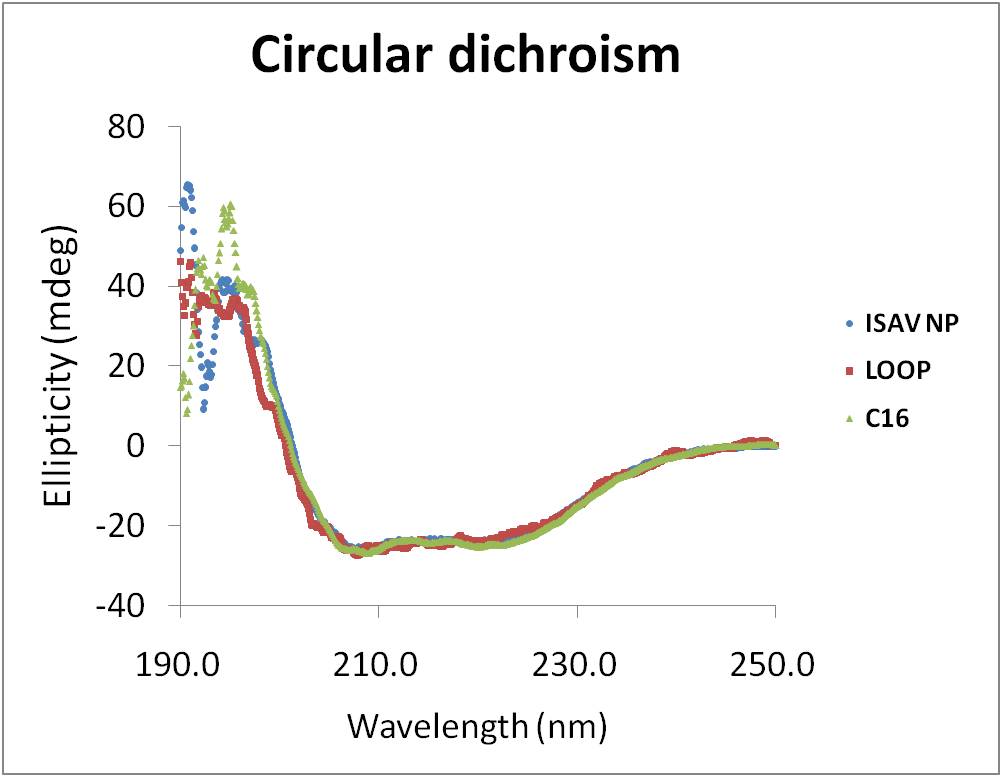


**Figure S5.** Far-UV CD Spectra of ISAV NP. All three NP proteins, including the *wt* NP (blue), ΔLOOP (red) and ΔC16 (green) were kept at 5 μM concentration. Protein buffer was 200 mM potassium phosphate pH 7.5. Measurements were done at room temperature.
