# Supplementary material for: The Crystal Structure and RNA-Binding of an Orthomyxovirus Nucleoprotein
Source: PLoS Pathog. 2013 Sep 12;9(9):e1003624. doi: 10.1371/journal.ppat.1003624 (PMC3771910; doi:10.1371/journal.ppat.1003624)
Supplement: Table S1 — Crystallographic data statistics. (DOCX) [file ppat.1003624.s006.docx]

**Table S1**. Crystallographic Data Statistics
